# Comparative genomic analysis of five coprinoid mushrooms species

**DOI:** 10.1007/s10142-023-01094-0

**Published:** 2023-05-13

**Authors:** Jingjing Wang, Ran Zhang, Guoao Ding, Lingling Wang, Wei Wang, Yan Zhang, GuiLan Zhu

**Affiliations:** 1 Department of Life Science, Hefei Normal University, Hefei, 230061 China; 2 Anhui Engineering Laboratory for Medicinal and Food Homologous Natural Resources Exploration, Hefei, 230061 China; 3 Department of Life Science, Anhui University, Hefei, 230601 China

**Keywords:** Fungal laccase, *Coprinus* species, Comparative genomic analysis, Gene family, Average nucleotide identity, Positive selection

## Abstract

Although coprinoid mushrooms are widely known for the phenomenon of deliquescence and production of fungal laccases and extracellular peroxygenases, the genome structure and genetic diversity of coprinoid mushroom species have not been extensively studied. To reveal the genomic structure and diversity in coprinoid mushroom species, the genomes of five coprinoid mushroom species were compared and analyzed. A total of 24,303 orthologous gene families, including 89,462 genes, were identified in the five species. The numbers of core, softcore, dispensable, and private genes were 5617 (25.6%), 1628 (7.4%), 2083 (9.5%), and 12,574 (57.4%), respectively. Differentiation time analysis revealed that *Coprinellus micaceus* and *Coprinellus angulatus* differentiated approximately 181.0 million years ago. *Coprinopsis cinerea* and *Coprinopsis marcescibilis* differentiated approximately 131.0 million years ago, and they were differentiated from *Candolleomyces aberdarensis* approximately 176.0 million years ago. Gene family contraction and expansion analyses showed that 1465 genes and 532 gene families were expanded, and 95 genes and 134 gene families were contracted. Ninety-five laccase-coding genes were detected in the five species, and the distribution of the laccase-coding genes in the five species was not uniform. These data provide a reference for a deeper understanding of the genetic structure of the genomes of coprinoid mushroom species. Furthermore, this study provides a reference for follow-up studies on the genome structure of coprinoid mushroom species and the diversity of specific functional genes.

**Supplementary Information:**

The online version contains supplementary material available at 10.1007/s10142-023-01094-0.

## Background

*Coprinus* species are cosmopolitan (Padamsee et al. 2008) and widely renowned for a phenomenon called deliquescence (Nagy et al. 2010). During this process, fruiting body tissues become blackish inky fluid by autodigestion of the fruiting body cells upon maturation (Hopple and Vilgalys 1999). Another notable feature of *Coprinus* is that most species can produce fungal laccases (Hoegger et al. 2004; Kilaru et al. 2006; Lin et al. 2013; Hu et al. 2019) and extracellular peroxygenases (Aranda et al. 2009). Laccases are biologically important enzymes that belong to the oxidase group and are useful as green enzymes for cleaner industrial applications to reduce environmental pollution (Senthivelan et al. 2016). Considering that these enzymes have various substrate catalytic properties and numerous applications in various fields, they have received attention from researchers for their use in further intensive studies worldwide (Senthivelan et al. 2016). Although laccases are produced by plants, bacteria, insects, and fungi (Dwivedi et al. 2011; Senthivelan et al. 2016), fungal laccases have been the most extensively studied (Mougin et al. 2003; Liu et al. 2022). However, the distribution of deliquescence and laccase- and peroxygenase-coding genes in *Coprinus* species does not exhibit a clear pattern, which may be due to the unclear classification system of *Coprinus* and the relatively complex genomes of *Coprinus* species (Nagy et al. 2010; Örstadius et al. 2015).

Traditionally, Psathyrellaceae species have been classified into two large genera, *Coprinus* and *Psathyrella*. However, the two genera have many common features, including similar habitat preferences, spore characteristics, degradation of spore pigments in sulfuric acid, and developmental, morphological, and ecological traits that are significantly convergent; several alternative classifications have also been proposed (Nagy et al. 2010). Furthermore, it has long been recognized that deliquescent taxa within Psathyrellaceae do not form a monophyletic group (Walther et al. 2005; Vašutová et al. 2008). The genus *Coprinus* was proposed to be split into four genera (Redhead et al. 2001). Although some studies have added valuable information to our knowledge of the phylogeny of Psathyrellaceae (Nagy et al. 2010; Örstadius et al. 2015; Wächter and Melzer 2020). However, these studies were all based on the diversity analysis of one or several limited genes. Even though these studies provide substantial guidance for the study of the phylogeny of Psathyrellaceae, they fail to provide effective information to study the origin and maintenance mechanisms of Psathyrellaceae functional genes.

Comparative genomics provides an important technical means for studying the origin and maintenance of fungal genetic diversity (Ma et al. 2013; de Vries et al. 2017; Kiss et al. 2019; Zhang et al. 2020). However, the genome structure and genetic diversity of coprinoid mushroom species have not been extensively studied. Therefore, in this study, we aimed to reveal the genomic structure and genetic diversity of coprinoid mushroom species by comparing and analyzing the genomes of five coprinoid mushroom species, i.e., *Coprinellus angulatus*, *Coprinellus micaceus*, *Coprinopsis cinerea*, *Coprinopsis marcescibilis*, and *Candolleomyces aberdarensis*.

## Materials and methods

### Genomic data collection of Coprinus species

The published genomes of *Coprinus* species were searched for and retrieved from NCBI, and the following five fully annotated fungi of the genus were retrieved: *C. aberdarensis* (60.61 Mb), *C. angulatus* (59.3 Mb), *C. micaceus* (77.39 Mb), *C. cinerea* (36.19 Mb), and *C. marcescibilis bilis* (38.91 Mb). *T. mesenterica* was selected as the outgroup (Table S1).

### Gene family clustering and enrichment

Amino acid sequences were aligned using BLASTp version 2.6.0 (parameter -evalue 1e − 5 -outfmt 6) (Camacho et al. 2009). Gene family clustering was performed using OrthoFinder version 2.3.12 (parameter -M msa) (Emms and Kelly 2019) Functional annotation results of GO (Ashburner et al. 2000) and Kyoto Encyclopedia of Genes and Genomes (KEGG) (Kanehisa and Goto 2000) were shown using the R clusterProfiler package (Wu et al. 2021). The numbers of non-redundant core, softcore, dispensable, and private gene families in each species and all species were counted.

### Analysis core and private gene families

A Venn diagram was drawn using the Perl script. Genes common to all genomes were defined as core genes, those common to 90% or more genomes were defined as softcore genes, those private to each genome were defined as private genes, and the remaining genes were defined as dispensable genes. Core and private gene analyses were performed using the R clusterProfiler package (Wu, Hu, Xu, Chen, Guo, Dai, Feng, Zhou, Tang, Zhan, Fu, Liu, Bo, and Yu 2021) based on the private gene and functional annotation results of GO (Ashburner et al. 2000) and KEGG (Kanehisa and Goto 2000).

### Phylogeny analysis and calculation of differentiation time

Multiple sequence alignment of protein sequences was performed using MUSCLE version 3.8.31 (Edgar 2004). The alignment sequences were filtered using trimAI v1.4. rev22 (parameter -gt 0.2) (Capella-Gutiérrez et al. 2009). The filtered alignment sequences were merged into supergenes. Finally, an maximum likelihood (ML) phylogenetic tree was constructed based on the supergenes using RAxML version 8.2.10 (Stamatakis 2014) with the PROTGAMMAWAG model.

The fossil time can make the calculation result of the differentiation time more accurate. Fossil timetables were obtained from TIMETREE (http://www.timetree.org/). Based on the topological structure of the phylogenetic tree and the fossil timetable, the differentiation times of the species were estimated using the mcmtree subprogram (parameters *n*sample = 3,000,000; burnin = 8,000,000; seqtype = 0; model = 4) of PAML version 4.9 (Yang 2007).

### Gene family contraction and expansion

The number of gene family members of the ancestors of each branch was estimated using the birth-mortality model based on the species evolutionary tree and gene family clustering results through café version 3.1 (Han et al. 2013), thereby the contraction and expansion of the gene family of the species relative to the ancestors were predicted.

### Analysis of laccase synthesis gene family

Laccase gene family numbers were searched on the InterPro website (https://www.ebi.ac.uk/interpro/result/InterProScan/), and PF numbers PF00394, PF07731, and PF07732 were obtained. Gene IDs were searched according to the PF numbers, and protein sequences were obtained according to the gene IDs. An ML phylogenetic tree based on laccase protein sequences was constructed as described above. Motif locations in laccase protein sequences were identified using MEME version 5.4.1 (Bailey and Elkan 1994).

### Genome average nucleotide identity analysis

ANI indicates the similarity of all orthologous protein-coding genes between two genomes and is often used to indicate the evolutionary distance between genomes (Pritchard et al. 2016). ANI was calculated using the pyani ANIm algorithm (https://pureportal.strath.ac.uk/en/publications/pyani-v028-average-nucleotide-identity-ani-and-related-measures-f).

## Results

### Gene family clustering and enrichment

**Fig. 1:**
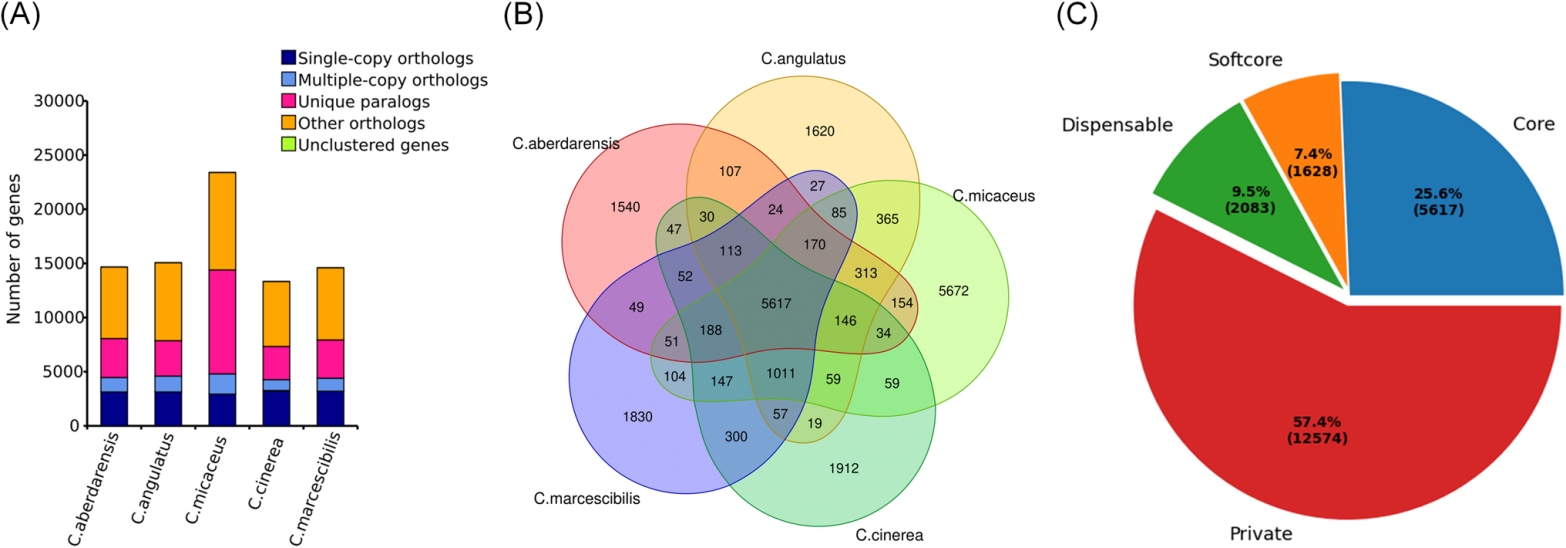
The number of homologous genes (**A**), core and private genes (**B**), and the number of genes of each classification (**C**). Genes common to all genomes were defined as core genes, those common to 90% or more genomes were defined as softcore genes, those private to each genome were defined as private genes, and the remaining genes were defined as dispensable genes

**Fig. 2:**
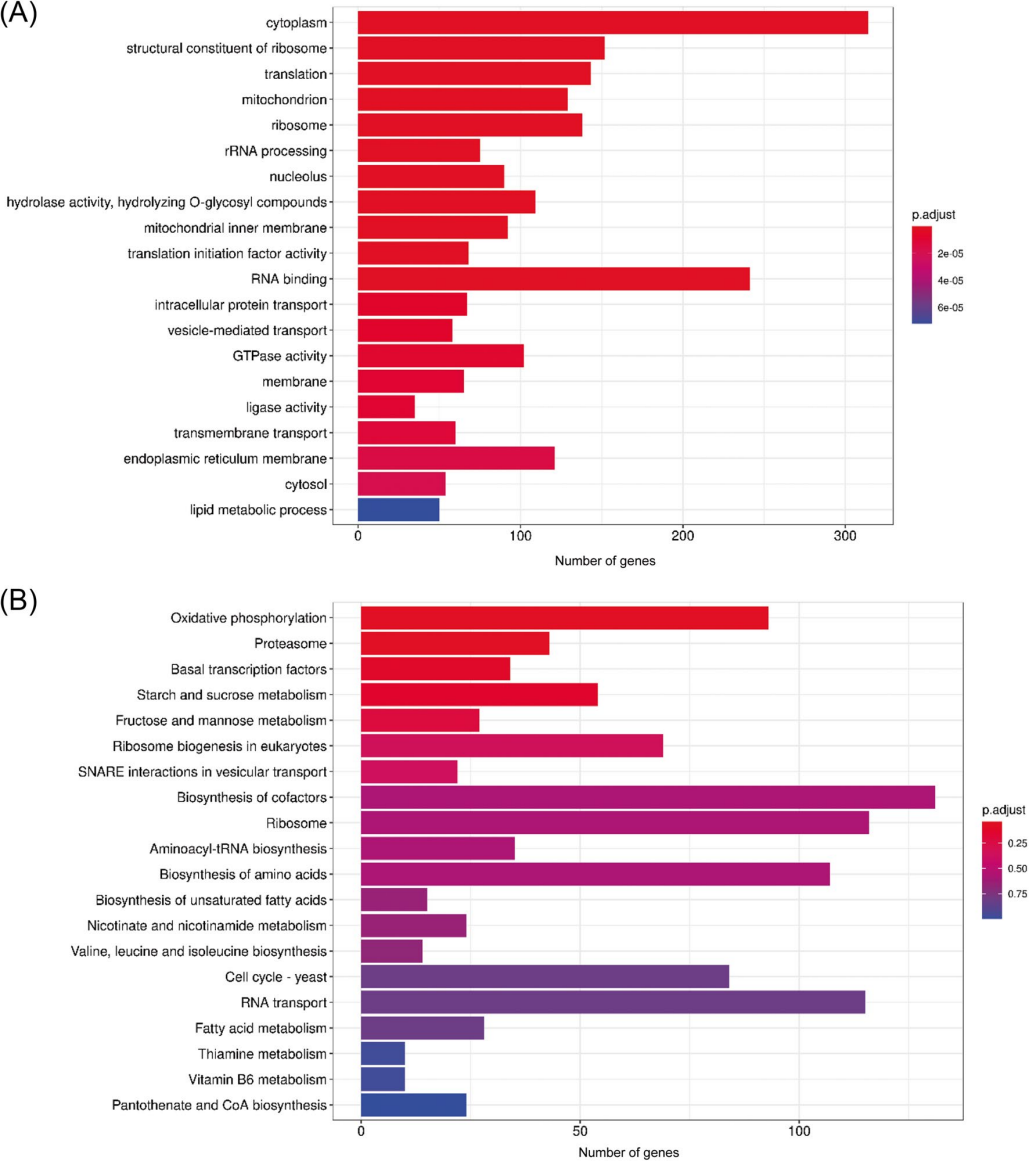
Gene ontology (**A**) and Kyoto ontology (**B**) enrichment of core genes

**Fig. 3:**
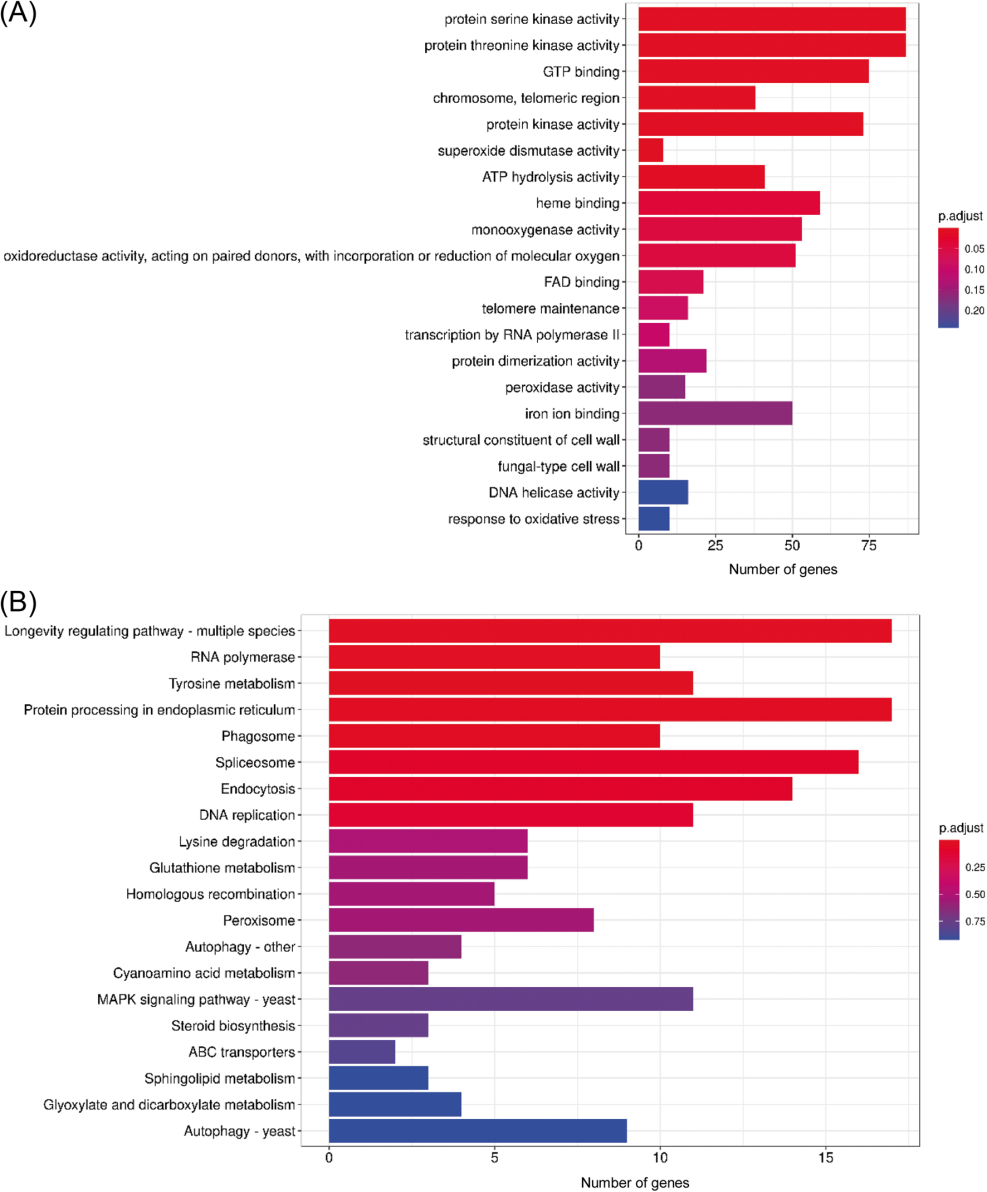
Gene ontology (**A**) and Kyoto ontology (**B**) enrichment of private genes

Five available genomic datasets from five *Coprinus* species were collected for comparative genome analysis. The genome size of the five species ranged from 36.19 to 77.39 Mb. A total of 21,902 orthologous gene families were identified, including 89,462 genes (Fig. 1A). The number of single-copy gene families was relatively stable in different species, with an average copy number of 3134.8 ± 58.98 (Fig. 1A). In this study, 5668 species-specific gene families were identified. Notably, in *C. micaceus*, the number of paralogous genes was markedly higher than in other species, reaching 5672 private genes (Fig. 1A and B). The numbers of core, softcore, dispensable, and private genes were 5617 (25.6%), 1628 (7.4%), 2083 (9.5%), and 12,574 (57.4%), respectively (Fig. 1C and Table S2). Kyoto ontology (KO) and gene ontology (GO) enrichment results showed that the core genes were primarily involved in regulating energy metabolism, biomass synthesis, and metabolic processes such as translation, mitochondrion, ribosome, rRNA processing, oxidative phosphorylation, biosynthesis amino acids, and fatty acid metabolism (Fig. 2). However, the private genes were primarily involved in some kinase, superoxide dismutase, monooxygenase, oxidoreductase, and peroxidase activities, structural constituents of the cell wall, and response to oxidative stress, which endowed fungi with the ability to participate in metabolism of substances such as tyrosine, glutathione, sphingolipid, glyoxylate, and dicarboxylate, and adapt to different habitats (Fig. 3).

### Phylogenetic tree, differentiation time, and gene family contraction and expansion

**Fig. 4:**
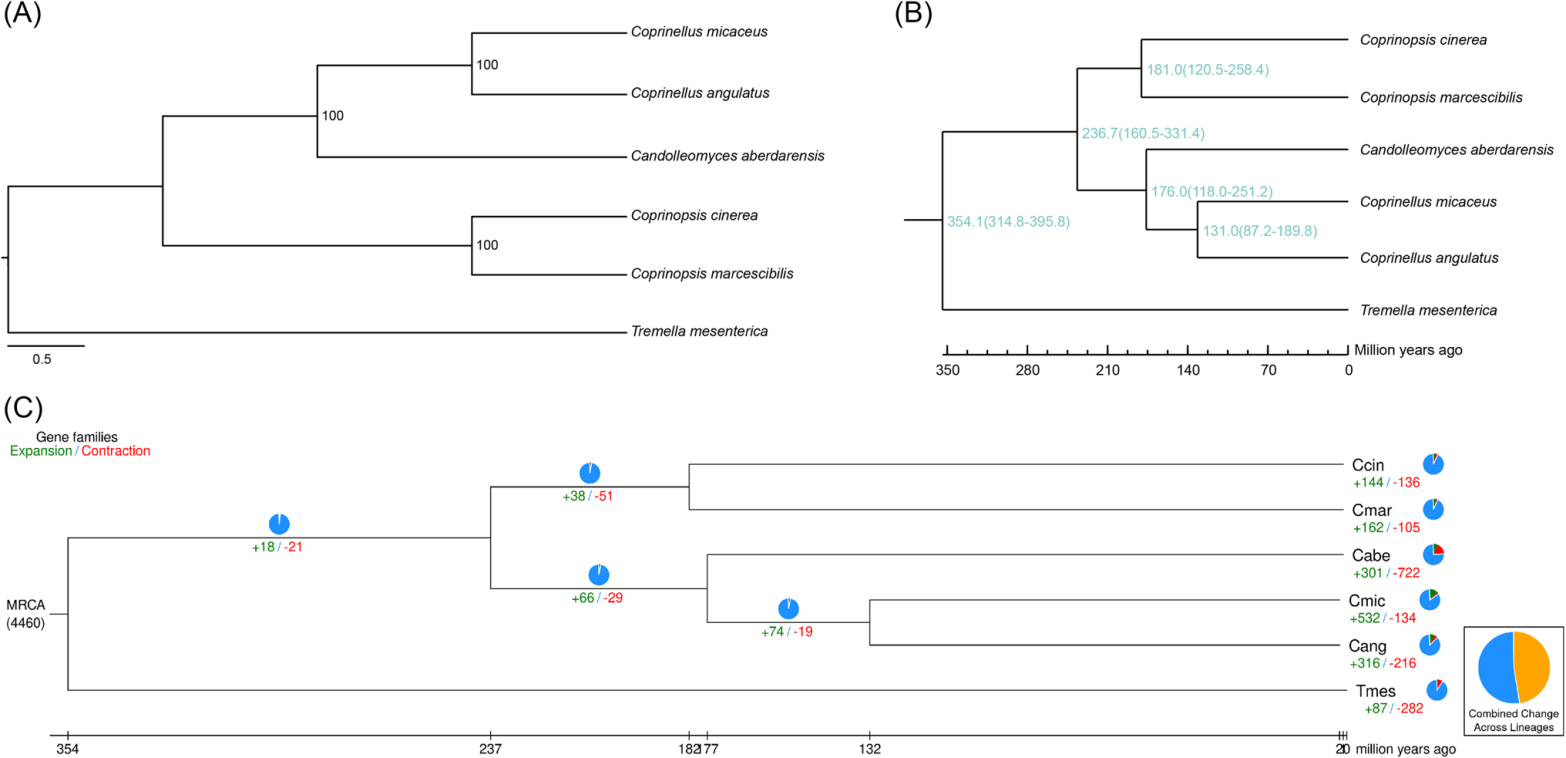
Phylogenetic tree (**A**), differentiation time (**B**), and gene family contraction and expansion (**C**). Ccin, *Coprinopsis cinerea*; Cmar, *Coprinopsis marcescibilis*; Cabe, *Candolleomyces aberdarensis*; Cmic, *Coprinellus micaceus*; Cang, *Coprinellus angulatus*; Tmes, *Tremella mesenterica*. Maximum likelihood phylogenetic tree was constructed based on the supergenes using RAxML version 8.2.10 with the PROTGAMMAWAG model. Fossil timetables were obtained from TIMETREE (http://www.timetree.org/). Based on the topological structure of the phylogenetic tree and the fossil timetable, the differentiation times of the species were estimated using the mcmtree subprogram (parameters *n*sample = 3,000,000; *b*urnin = 8,000,000; seqtype = 0; model = 4) of PAML version 4.9

The phylogenetic tree constructed using 2353 single-copy genes showed that *C. micaceus* and *C. angulatus* were clustered into one branch and then clustered with *C. aberdarensis*. *C. cinerea* and *C. marcescibilis* were clustered into one branch. *Tremella mesenterica* was furthest from its evolution (Fig. 4A). Differentiation time analysis showed that *C. micaceus* and *C. angulatus* differentiated approximately 131.0 (from 87.2 to 189.8) million years ago, and they have differentiated from *C. aberdarensis* approximately 176.0 (from 118.0 to 251.2) million years ago. *C. cinerea* and *C. marcescibilis* differentiated approximately 181.0 (from 120.5 to 258.4) million years ago (Fig. 4B). However, genome average nucleotide identity (ANI) analysis showed that, as an outgroup, the ANI between *T. mesenterica* and *C. marcescibilis* was 1, and that between *T. mesenterica* and *C. angulatus* was 0.93, which was higher than that between *C. marcescibilis* and *C. cinerea*, as well as that between *C. micaceus* and *C. angulatus* (0.84; Table S3).

The results through café version 3.1 (Han et al. 2013) indicated that the expansion and contraction gene families in the *C. micaceus* genome were 532 and 134, respectively, in which the expansion gene families were more numerous than the contraction gene families. However, the expansion and contraction gene families in *C. aberdarensis* were 301 and 722, respectively, in which the expansion gene families were fewer than the contraction gene families (Fig. 4C). Compared with *Coprinopsis*, the genomes of the two *Coprinellus* species contained more expansion and contraction gene families (Fig. 4C). Furthermore, extraction and enrichment analysis of the contraction and expansion genes and gene families showed that 1465 genes and 532 gene families were expanded, and 95 genes and 59 gene families were contracted within species (Table S4).

**Fig. 5:**
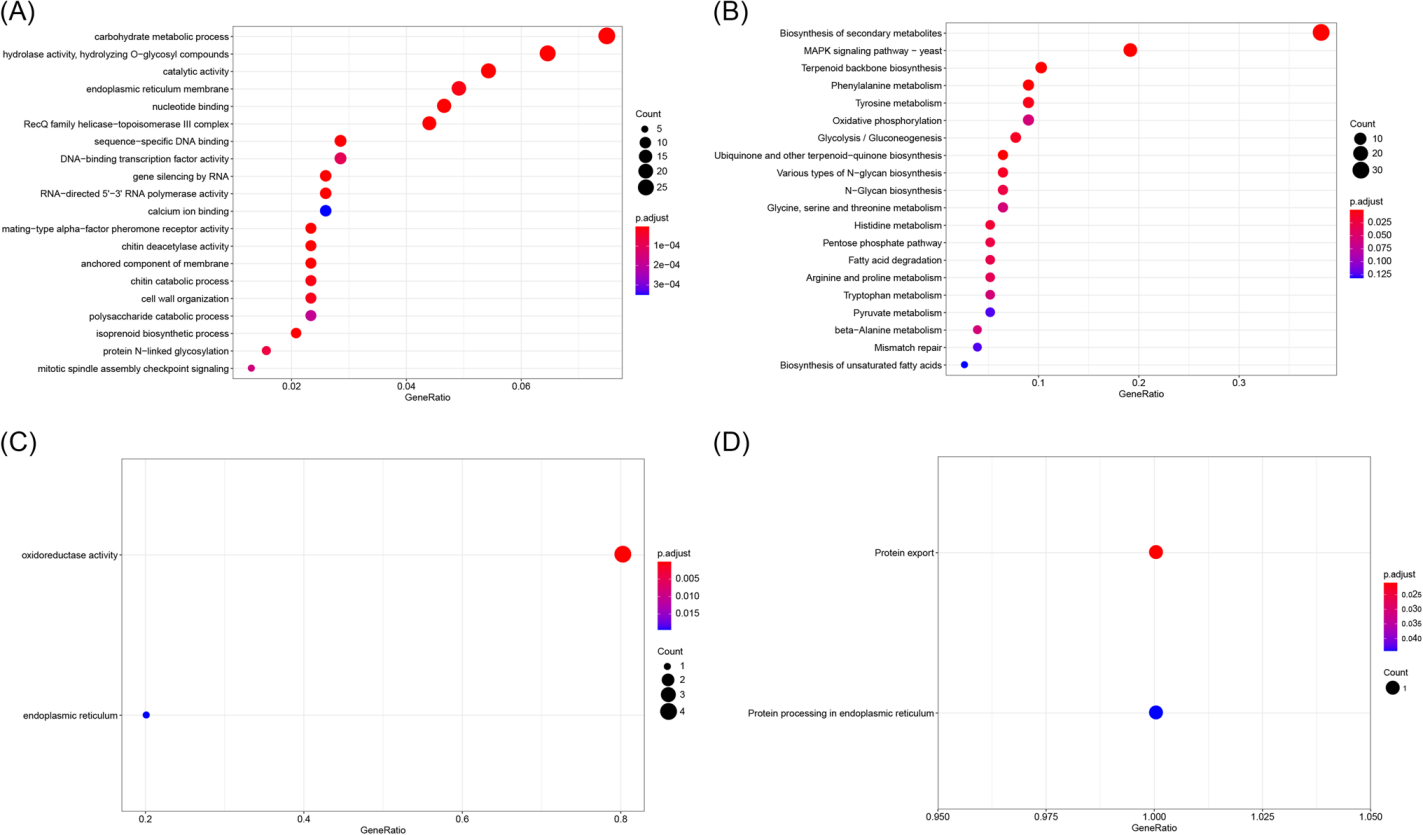
Gene ontology (GO) and Kyoto Encyclopedia of Genes and Genomes ontology (KO) enrichment of expansion and contraction gene families. **A**–**D** The GO and KO enrichment of expansion gene families and GO and KO enrichment of contraction gene families, respectively. The diameters of the circle indicate the number of genes

The GO and KO enrichment results showed that the expansion genes mainly participated in carbohydrate metabolic processes, catalytic activity, and catabolic processes, which endowed the fungi with the ability to synthesize and metabolize substances and adapt to different habitats (Fig. 5). The contraction genes exhibited oxidoreductase activity and participated in protein processing in the endoplasmic reticulum and export (Fig. 5).

### Laccase gene family

**Fig. 6:**
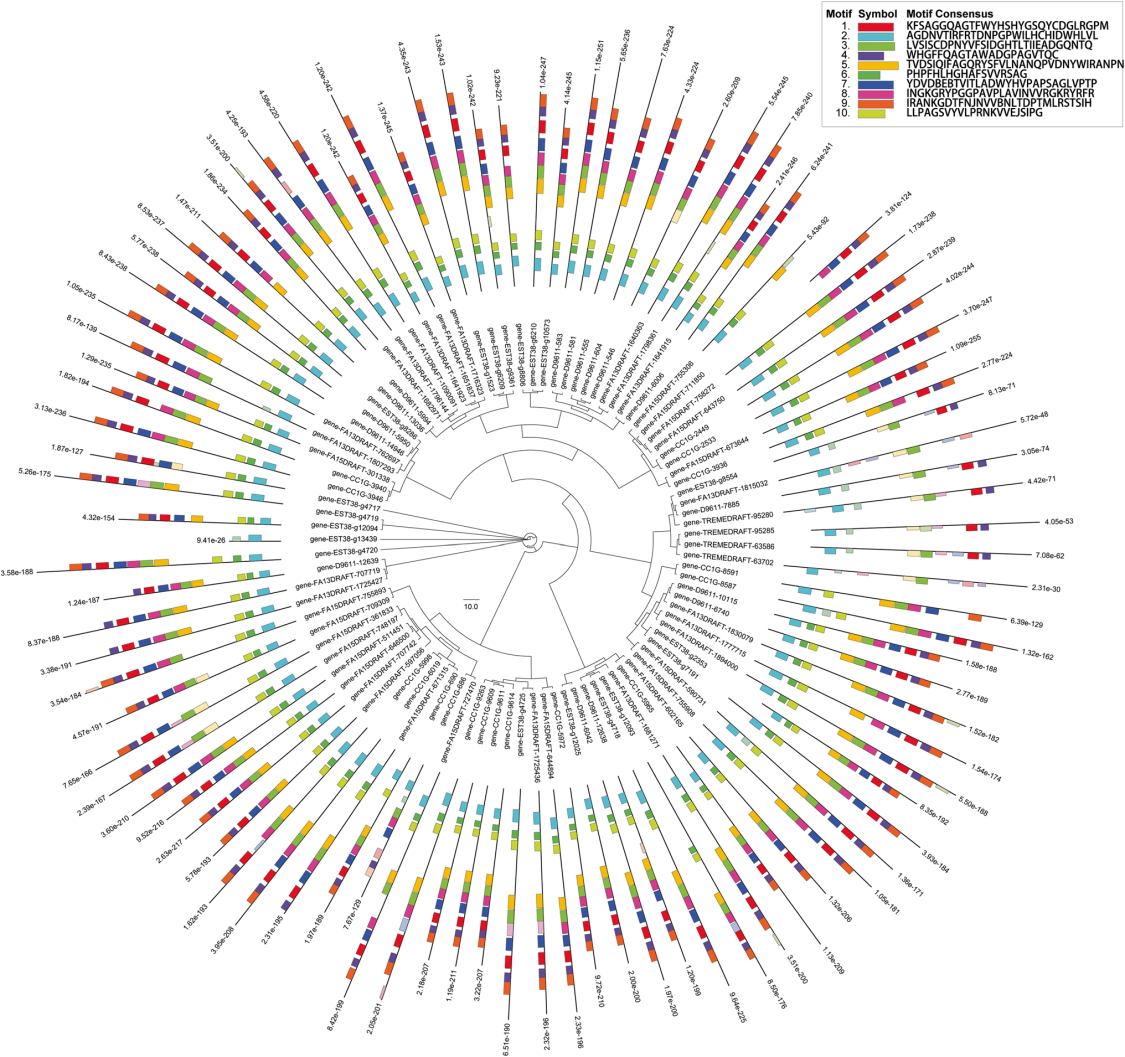
Phylogenetic tree of laccase genes and motif distribution in the laccases. The motif sites were predicted by MEME plus any additional sites detected using a motif scanning algorithm. The MEME sites are shown in solid color, and additional scanned sites are shown in transparent color. Hovering the cursor over a site will reveal details about the site. Only sequences containing a predicted or scanned motif site are shown. The scanned sites are predicted using a log-odds scoring matrix constructed from the MEME sites. Only scanned sites with position *p*-values less than 0.0001 are shown

Ninety-five laccase genes were detected in the five species (Fig. 6). These genes were highly diverse (Fig. 6). Most laccase genes contain 10 motifs in a similar order (Fig. 6). However, the distribution of the laccase genes among the five species was not uniform. The *C. micaceus* genome contained the most multicopper oxidase PF07731 genes, the *C. marcescibilis* genome contained the most multicopper oxidase PF00394 genes, and the *C. aberdarensis* genome contained the most multicopper oxidase PF07732 genes (Fig. S1).

## Discussion

With the in-depth development of sequencing technology and bioinformatics analysis tools, comparative genomics has been widely used in studies on species evolution and formation, ecological diffusion, habitat adaptation, and antibiotic resistance gene diffusion (Ni 2016; Lin et al. 2018; Qin et al. 2021; Shah et al. 2022; Wang et al. 2022; Yao et al. 2022). The core genome is generally considered the minimum genome necessary for the survival of free-living organisms (Zafar et al. 2002; Pandaranayaka et al. 2019). Therefore, core genes are closely related to the metabolic processes necessary for survival. Private genes are usually related to the secondary metabolites of fungi and enable different fungi to synthesize different small molecules and adapt to different habitats (Wisecaver et al. 2014; Keller 2019; Pandaranayaka et al. 2019; Slot and Gluck-Thaler 2019). Our results indicate that the core genes were mainly involved in the regulation of energy metabolism, biomass synthesis, and metabolic processes, whereas the private genes mainly involved some kinase, superoxide dismutase, monooxygenase, oxidoreductase, and peroxidase activities, structural constituents of the cell wall, and response to oxidative stress, which endowed fungi with the ability to participate in substance metabolisms, such as tyrosine, glutathione, sphingolipid, glyoxylate, and dicarboxylate, and adapt to different habitats. These results indicate that private genes endow different fungi with different secondary metabolic characteristics and habitat adaptability, which is of substantial value for maintaining fungal biodiversity. Notably, the expansion genes mainly participated in carbohydrate metabolic processes, catalytic activity, and catabolic processes, which endowed the fungi with the ability to synthesize and metabolize secondary metabolites and adapt to different habitats. These results imply that fungal differentiation is primarily due to the differentiation of private genes. Considering that secondary metabolites are mainly produced by private genes, it may be of considerable significance to use secondary metabolites of fungi as a basis for fungal classification.

As sequencing technology has advanced, fungal classification has shifted from morphological characteristics to molecular phylogenetic evidence (Nagy et al. 2010; Örstadius et al. 2015). Recently, Wächter and Melzer (2020) inferred that the family Psathyrellaceae forms distinct phylogenetic clades and is divided into 16 genera based on molecular phylogenetic evidence and morphological characteristics (Wächter and Melzer 2020). Although some morphological features caused by private genes may be used as the basis for fungal classification, the secondary metabolites produced by private genes are difficult to reflect in morphology, so it is difficult to use them for fungal morphological classification. Furthermore, genome ANI analysis showed that, as an outgroup, the ANI between *T. mesenterica* and *C. marcescibilis* was 1 and that between *T. mesenterica* and *C. angulatus* was 0.93, which was higher than that between *C. marcescibilis* and *C. cinerea*, as well as that between *C. micaceus* and *C. angulatus*. These results indicate that it is difficult to classify fungi effectively using only a few genes. Therefore, it is necessary to identify secondary metabolites for fungal classification.

Laccases, involved in decomposing various aromatic compounds, such as lignin and humic matter, are ubiquitously distributed in nature, such as plants, fungi, and bacteria (Luo et al. 2015; Moreno et al. 2017; Janusz et al. 2020). Laccase activity revealed the linkage between fungal laccase genes and the fate of soil organic matter in ecosystems, and the diversity and richness of fungal laccase genes decreased with soil organic matter concentration (Kellner et al. 2009; Theuerl and Buscot 2010). Moreover, laccases have often been associated with fungal infection of plant and animal hosts (Moreno et al. 2017). Generally, most fungi possess multiple copies of genes encoding laccases and producing several laccase isoenzymes, which implies that these enzymes perform a variety of physiological functions (Luis et al. 2004; Moreno et al. 2017). Characterization of laccase families in fungi requires attention to elucidate their exact functional relationships (Moreno et al. 2017). Twenty-six genes were extracted in five clinical and environmental *Fonsecaea* species, and those genes possess features used as evidence of functional laccases (Moreno et al. 2017). Our results indicated that 95 laccase genes were detected in the five *Coprinus* species, which were evidently higher than the five clinical and environmental *Fonsecaea* species (Moreno et al. 2017). These results implied that the diversity of laccase genes possibly plays a crucial role in the survival of *Coprinus* species.

## Conclusions

A total of 24,303 orthologous gene families were identified in the five *Coprinus* species, including 89,462 genes. The core genes were mainly involved in the regulation of energy metabolism, biomass synthesis, and metabolic processes, whereas the private genes endowed the fungi with the ability to participate in substance metabolism and adapt to different habitats. These results indicate that private genes endow different fungi with different secondary metabolic characteristics and habitat adaptability, which is of considerable importance for maintaining fungal biodiversity. These results imply that fungal differentiation is mainly due to the differentiation of private genes. Considering that secondary metabolites are primarily produced by private genes, using secondary metabolites of fungi as a basis for fungal classification may be of great significance.

## Supplementary Information

Below is the link to the electronic supplementary material.

Supplementary file1 (DOCX 34 KB)

Supplementary file2 (XLSX 48 KB)

## Data Availability

Not applicable.
