# Supplementary material for: Comparative genomic analysis of five coprinoid mushrooms species
Source: Funct Integr Genomics. 2023 May 13;23(2):159. doi: 10.1007/s10142-023-01094-0 (PMC10182949; doi:10.1007/s10142-023-01094-0)

**Supplementary Materials**

Table S1. Website of genome source.

| Species | Website |
| --- | --- |
| *Coprinellus angulatus* | https://www.ncbi.nlm.nih.gov/genome/?term=Coprinellus+angulatus |
| *Coprinellus micaceus* | https://www.ncbi.nlm.nih.gov/genome/?term=Coprinellus+micaceus |
| *Coprinopsis cinerea* | https://www.ncbi.nlm.nih.gov/genome/?term=Coprinopsis+cinerea |
| *Coprinopsis marcescibilis* | https://www.ncbi.nlm.nih.gov/genome/?term=Coprinopsis+marcescibilis |
| *Candolleomyces aberdarensis* | https://www.ncbi.nlm.nih.gov/genome/?term=Candolleomyces+aberdarensis |
| *Tremella mesenterica* | https://www.ncbi.nlm.nih.gov/genome/?term=Tremella+mesenterica |

Table S2. Number of genes in different classifications

| Species | Core | Softcore | Dispensable | Private | Total |
| --- | --- | --- | --- | --- | --- |
| *Candolleomyces aberdarensis* | 5,617 | 617 | 861 | 1,540 | 1,540 |
| *Coprinellus angulatus* | 5,617 | 1,440 | 1,086 | 1,620 | 1,620 |
| *Coprinellus micaceus* | 5,617 | 1,515 | 1,371 | 5,672 | 5,672 |
| *Coprinopsis cinerea* | 5,617 | 1,458 | 804 | 1,912 | 1,912 |
| *Coprinopsis marcescibilis* | 5,617 | 1,482 | 896 | 1,830 | 1,830 |

Table S3. Genome ANI results between six fungi species

|  | *Candolleomyces aberdarensis* | *Coprinopsis marcescibilis* | *Coprinellus micaceus* | *Coprinellus angulatus* | *Coprinopsis cinerea* | *Tremella mesenterica* |
| --- | --- | --- | --- | --- | --- | --- |
| *Candolleomyces aberdarensis* | 1 | 0.85 | 0.84 | 0.84 | 0.85 | 0.81 |
| *Coprinopsis marcescibilis* | 0.85 | 1 | 0.84 | 0.84 | 0.84 | 1 |
| *Coprinellus micaceus* | 0.84 | 0.84 | 1 | 0.84 | 0.85 | 0.84 |
| *Coprinellus angulatus* | 0.84 | 0.84 | 0.84 | 1 | 0.85 | 0.93 |
| *Coprinopsis cinerea* | 0.85 | 0.84 | 0.85 | 0.85 | 1 | 0.82 |
| *Tremella mesenterica* | 0.81 | 1 | 0.84 | 0.93 | 0.82 | 1 |

Table S4. Number of contraction and expansion gene families and genes

| Type | Expansion | Contraction |
| --- | --- | --- |
| Gene | 1,465 | 95 |
| Gene family | 532 | 59 |

Figure S1. Distribution of laccase coding gene families in the species. The values corresponding to the heatmap colors are converted by Z-score.


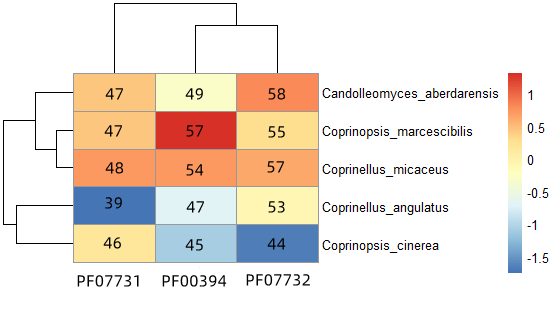

Supplement: Supplementary file 1 — Supplementary file1 (DOCX 34 KB) [file 10142_2023_1094_MOESM1_ESM.docx]
